# Supplementary material for: De-Suppression of Mesenchymal Cell Identities and Variable Phenotypic Outcomes Associated with Knockout of Bbs1
Source: Cells. 2023 Nov 20;12(22):2662. doi: 10.3390/cells12222662 (PMC10670506; doi:10.3390/cells12222662)

Supplementary Table S1.

sgRNA	Transfection efficiency (% GFP-positive cells, determined by FACS)	No. wells seeded by single-cell sort	No. colonies established after single-cell sort (as a % of total wells seeded with single cells)	No. clones expanded	No. clones genotyped	No. clones with biallelic indels (as a % of clones genotyped)	No. heterozygous clones (as a % of clones genotyped)	No. WTcr clones (as a % of clones genotyped)
sgRNA-1	43.4 %	288	67 (23.3 %)	14	6	4 (66.7 %)	0 (0 %)	2 (33 %)
sgRNA-8	44.4 %	288	90 (31.2 %)	16	6	3 (50.0 %)	0 (0 %)	3 (50 %)
sgRNA-12	46.3 %	96	34 (35.4 %)	34	32	14 (43.8 %)	0 (0 %)	18 (56 %)
**Total (Mean)**	**(44.7 %)**	**672**	**191 (28.4 %)**	**64**	**44**	**21 (47.7 %)**	**1 (1.7 %)**	**32 (55.3 %)**

Supplementary Table S2.

Clone name	Mutation 1	Mutation 2	Mutation 3
Bbs1$^{ex1.1}$	c.1_4del	c.4del	-
Bbs1$^{ex1.2}$	c.1_4del	c.4del	-
Bbs1$^{ex1.3}$	c.3_4del	c.3_7del	-
Bbs1$^{ex1.4}$	c.-16_3del	c.-16_3del	-
Bbs1^{ex8}	c.626del	c.626_627insA	-
Bbs1$^{ex12.1}$	c.1175_1176insC	c.1175_1179del	c.1175_1176insG
Bbs1$^{ex12.2}$	c.1175del	c.1175del	-
Bbs1$^{ex12.3}$	c.1175_1176insC	c.1166-1173del	-
Bbs1$^{ex12.4}$	c.1174-1180+4del	c.1175_1179del	c.1175_1176del
Bbs1^{M390R}	c.[1161C>A;1169T>G]	c.1154_1173del	-

Supplementary Figure S1.

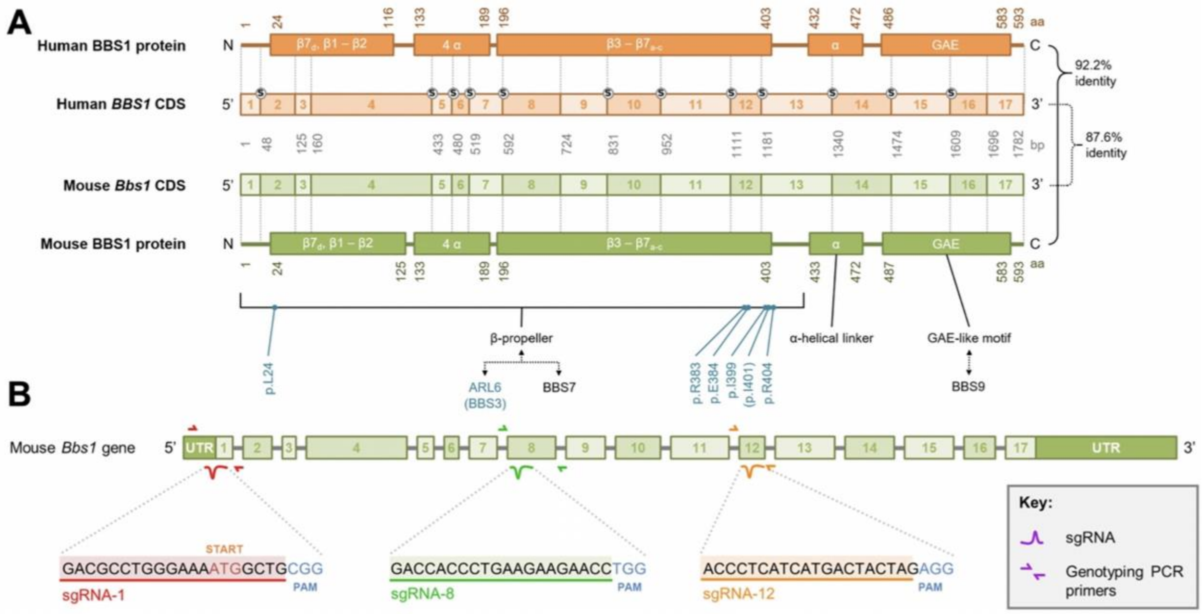

A. Alignment of mouse and human *Bbs1*/*BBS1* genes and proteins.
B. Map of RT-qPCR primer annealing sites relative to sgRNA target loci. Primers were designed to span an exon-exon junction at a site distal to the target sites of sgRNAs.

Supplementary Figure S2.

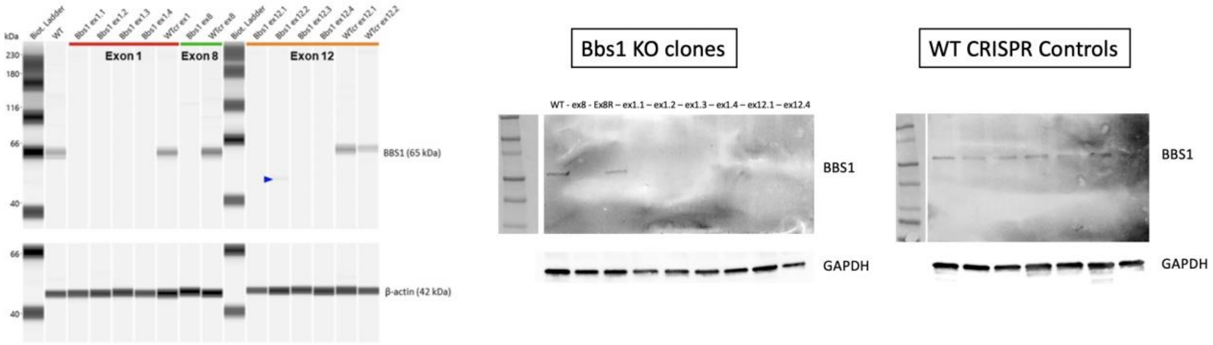

Capilliary based Western blot (PeggySue) using anti-BBS1 Abcam mAb identified 8 clones were true knockouts (no detectable protein at the expected molecular weight (65 kDa)). Protein of abnormal size was detected in Bbs1ex12.2 (48 kDa). These knockouts were confirmed with conventional Western blots and anti-β-actin antibody is used to demonstrate equal loading.

Supplementary Figure S3.

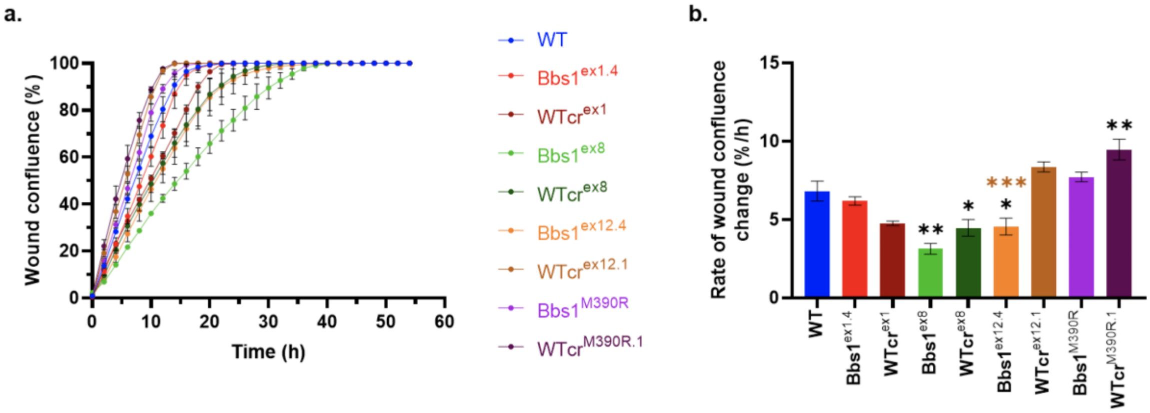

a. Wound healing over time, measured as the percentage cell confluence within the initial scratch wound area ("wound confluence"). Points show the mean wound confluence at 2-hour intervals over a 54-hour healing period. Error bars show the SEM of three independent experiments.

b. Rate of wound healing, measured as change in percentage confluence per hour (%/h), was extracted from the linear portions of wound healing graphs. Coloured bars show the mean rate of wound healing and error bars show the SEM of three independent experiments. One-way ANOVA indicated a significant difference among means of different cell lines (p<0.0001). Post-hoc Bonferroni multiple comparisons tests indicated that, compared to WT, Bbs1ex8 (padj=0.0002), WTcrex8 (padj<0.0207) Bbs1ex12.4 (padj=0.0278) and WTcrM390R.1 (padj=0.0072) differed significantly, whereas Bbs1ex1.4 (padj>0.9999), WTcrex1 (padj=0.0565), WTcrex12.1 (padj=0.3158) and Bbs1M390R (padj>0.9999) did not. Compared to their respective WTcr clones, Bbs1ex12.4 (padj=0.0001) differed significantly, whereas Bbs1ex1.4 (p-adj>0.4629), Bbs1ex8 (padj=0.6077) and Bbs1M390R (padj=0.1611) did not. On the graph, adjusted p-values are indicated: * padj=0.01 to 0.05; ** padj=0.001 to 0.01; *** p-adj<0.001; those in black are relative to WT and those in colour are relative to the respective WTcr clone.

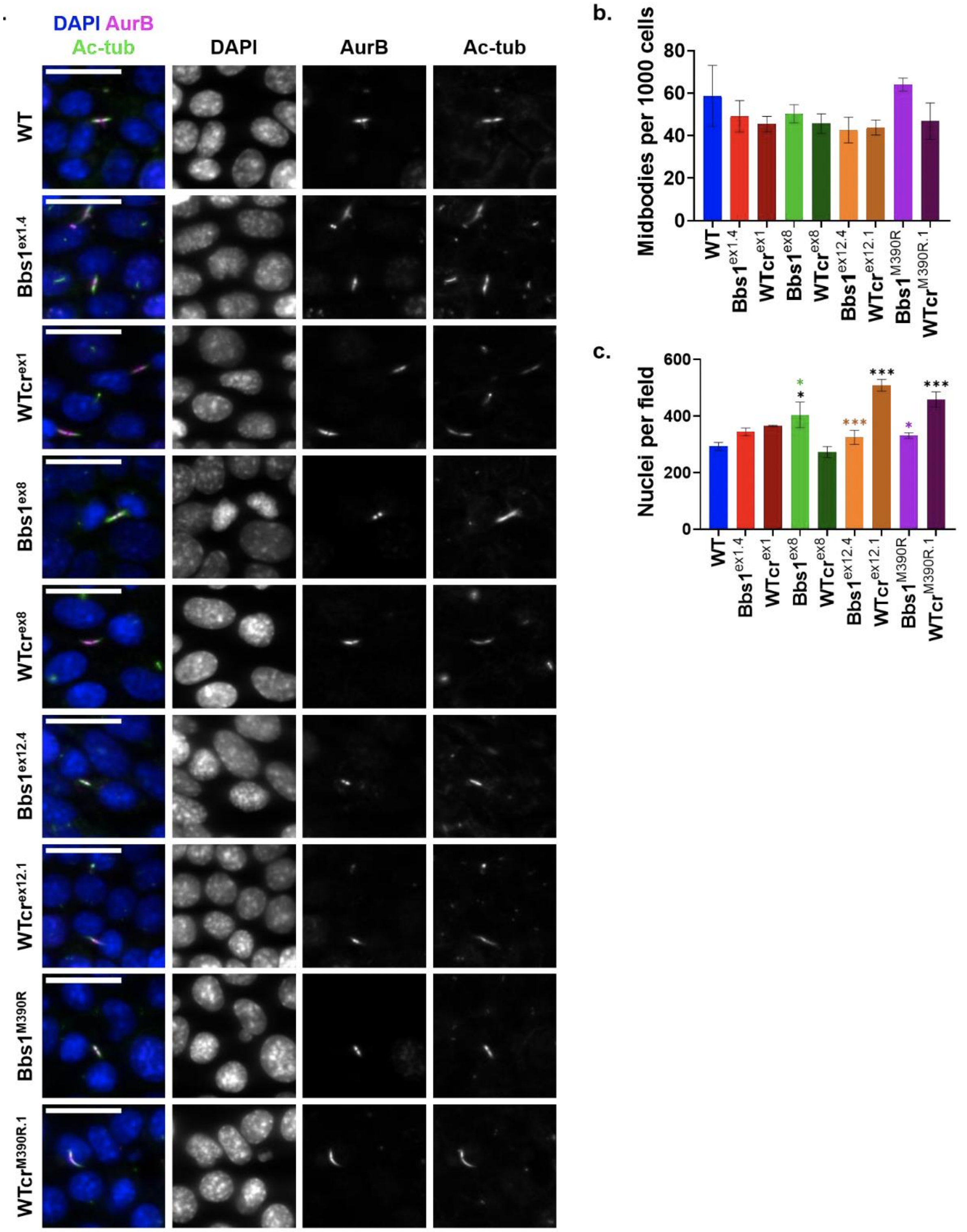

a. Representative staining of mid-bodies. A midbody-specific marker, Aurora B kinase (AurB), disting-uished midbodies from cilia, as both structures contain acetylated-α-tubulin (ac-tub). Nuclei were stained with DAPI. Z-projections of planes containing midbodies are displayed. Scale bars represent 20 µm.

b. Quantification of midbodies, given as the number of midbodies per 1000 cells. Coloured bars show the mean number of midbodies per 1000 cells and error bars show SEM of three independent experiments. One-way ANOVA showed that there was no statistically significant difference between the means of different cell lines ($p=0.4371$).

c. Mean number of nuclei per midbody assay image. Coloured bars show mean numbers of nuclei per field and error bars show SEM of three independent experiments. One-way ANOVA indicated a significant difference among means of different cell lines (p<0.0001). Post-hoc Bonferroni multiple comparisons tests indicated that, compared to WT, Bbs1ex8 (padj=0.0350), WTcrex12.1 (padj<0.0001) and WTcrM390R.1 (padj=0.0009) differed significantly, whereas Bbs1ex1.4 (padj>0.9999), WTcrex1 (padj=0.4636), WTcrex8 (padj>0.9999), Bbs1ex12.4 (padj>0.9999) and Bbs1M390R (padj>0.9999) did not. Compared to their respective WTcr clones, Bbs1ex8 (p-adj=0.0084), Bbs1ex12.4 (padj=0.0003) and Bbs1M390R (padj=0.0117) differed significantly, whereas Bbs1ex1.4 did not (padj>0.9999). On the graph, adjusted p-values are indicated: * padj=0.01 to 0.05; ** padj=0.001 to 0.01; *** padj<0.001; those in black are relative to WT and those in colour are relative to the respective WTcr clone.

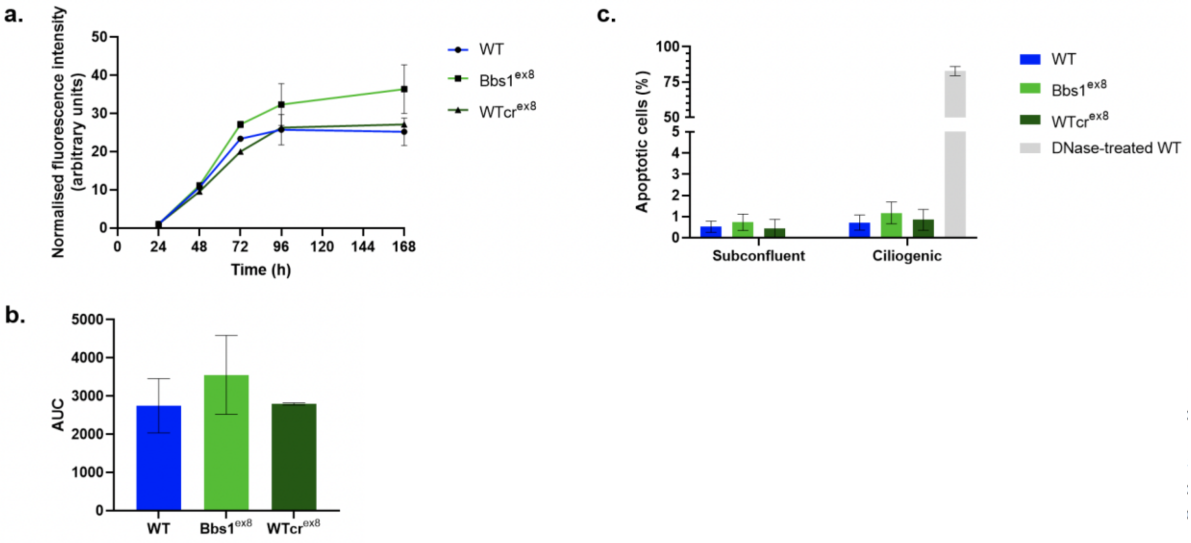

a. Fluorescence-based assay of cell viability. Resazurin (a weakly-fluorescent dye) is reduced to resorufin (a highly-fluorescent dye) by metabolically-active cells. Points show mean fluorescence intensity of resorufin normalised to the fluorescence intensity reading on day 1 (24 h). Error bars show SEM of three independent experiments. In Bbs1ex8 cultures, fluorescence intensity began to increase above wild type levels after 48 h.
b. Mean area under the curves (AUC) of cell viability time series. For each cell line, the AUC was calculated from each replicate time series. Coloured bars show the mean AUC and error bars show the SEM of three independent experiments. Although the AUC indicated increased fluorescence intensity in Bbs1ex8 cultures over the time series, paired t-tests showed it was not significantly different compared to WT (p=0.0537) or WTcrex8 (p=0.3249) cultures. There was also no statistically significant difference between WTcrex8 and WT (p=0.9200).
c. Quantification of percentage of apoptotic cells by TUNEL assay. DNase treatment of WT IMCD3 cells was used as a positive control. Coloured bars show the mean percentage of apoptotic (TUNEL-positive) cells and error bars show the SEM of three independent experiments, performed under subconfluent or ciliogenic conditions. Paired t-tests were performed after arcsine square root transformation of proportions of apoptotic cells. There was no statistically significant difference between the numbers of nuclei per field of pairs of cell lines, whether under subconfluent conditions (p=0.8675, p=0.5357 and p=0.5795, for Bbs1ex8 versus WT, Bbs1ex8 versus WTcrex8 and WTcrex8 versus WT, respectively), or

under ciliogenic conditions (p=0.4436, p=0.4651and p=0.8824, for Bbs1ex8 versus WT, Bbs1ex8 versus WTcrex8 and WTcrex8 versus WT, respectively).

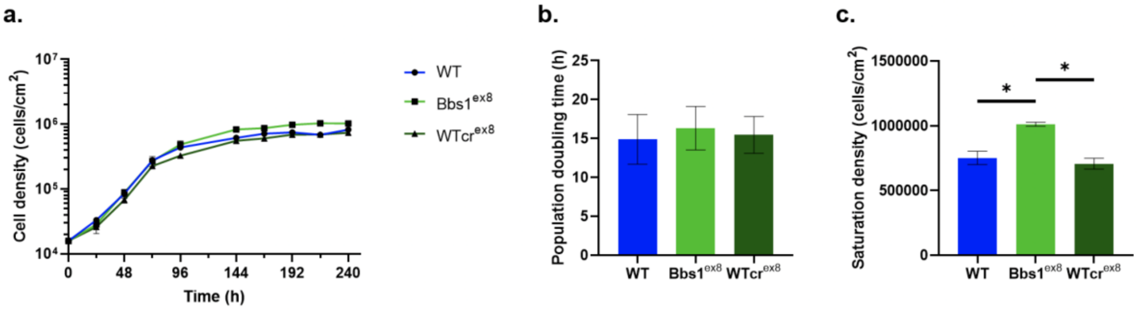

a. Growth curves of Bbs1ex8, WTcrex8 and WT IMCD3 cell lines. Points represent the mean cell density and error bars show the SEM of three independent experiments. Note in most cases the error bars are too small to be seen on a logarithmic scale.
b. Population doubling times (PDT) were extracted from the exponential growth phase of growth curves. Coloured bars show the mean PDT and error bars show the SEM of three independent growth curves. Paired t-tests indicated there was no statistically significant difference between the PDT of Bbs1ex8 and WT (p=0.1766) or WTcrex8 (p=0.4922), nor between WTcrex8 and WT (p=0.4904).
c. Saturation densities were extracted from the plateau phase of growth curves. Coloured bars show the mean saturation density and error bars show the SEM of three independent growth curves. Paired t-tests indicated that Bbs1ex8 had a significantly increased saturation density compared to WT (p=0.0194) and WTcrex8 (p=0.0119). There was no significant difference in the saturation densities of WTcrex8 and WT (p=0.1716). P-values from t-tests are indicated: * p=0.01 to 0.05; ** p=0.001 to 0.01; *** p< 0.001.

Supplementary Figure S4.

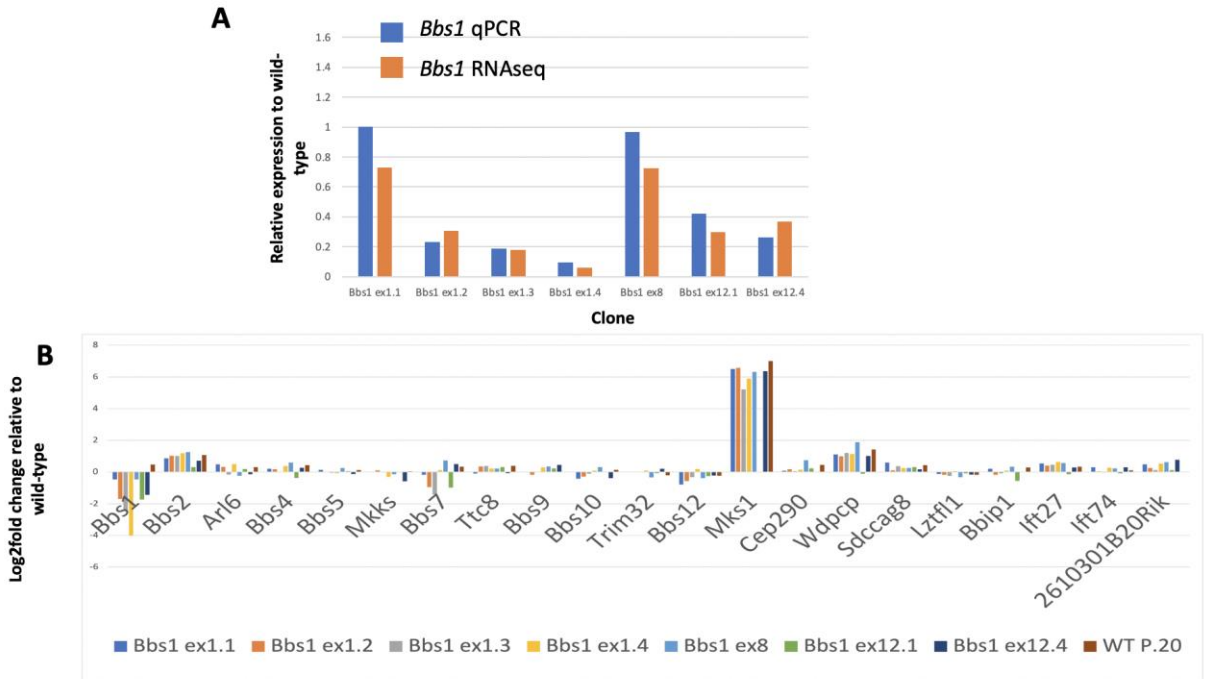

Bbs gene expression in the indicated clones relative to passage matched parental wild-type.

Supplementary Figure S5.

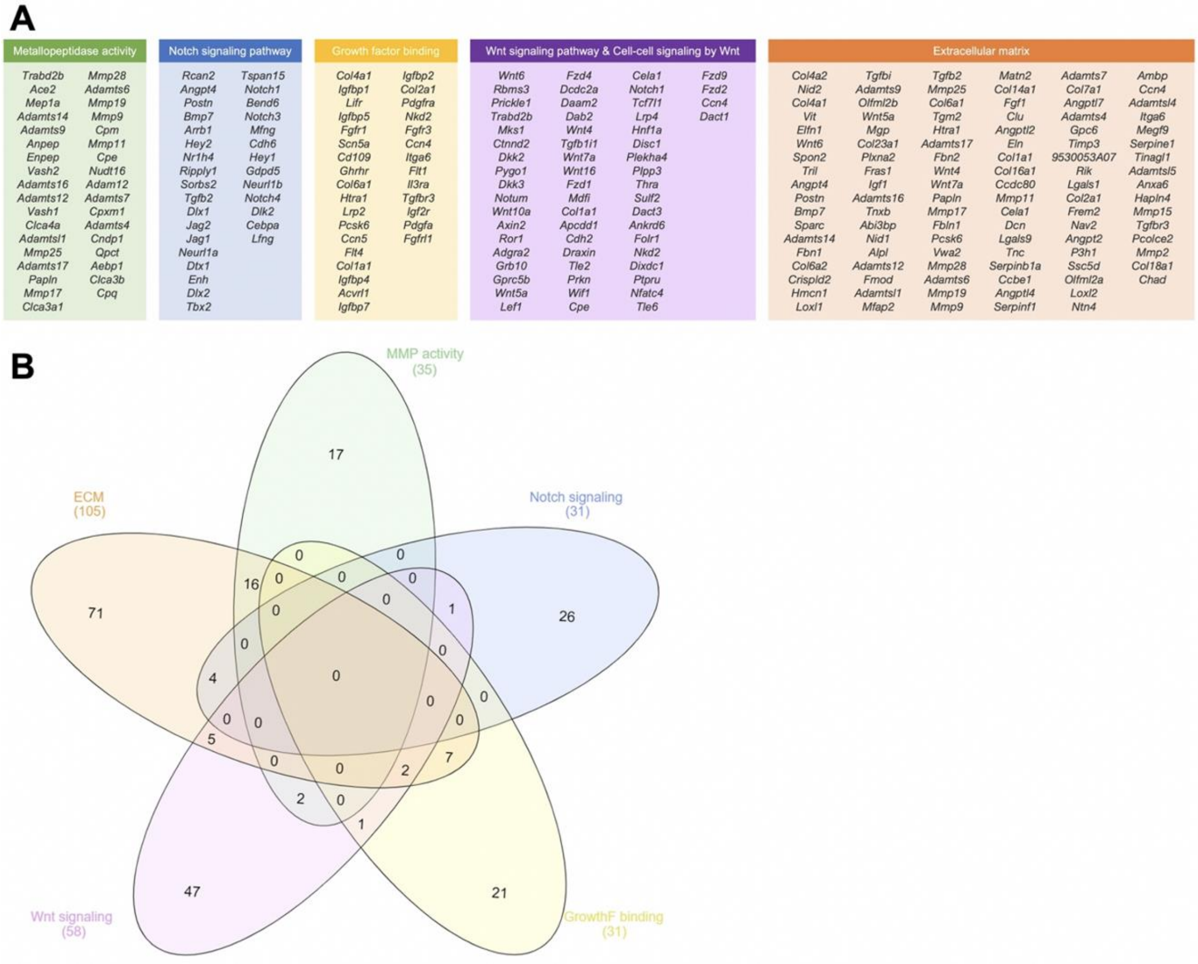

The four sources of cilia genes are displayed in a Venn diagram. These were: the SysCilia Gold Standard (SCGS) and SCGS potential ciliary genes, CiliaCarta Bayesian predictions and Gene Ontology cellular component (GO CC) terms associated with the cilium. The Venn diagram indicates the numbers of orthologous mouse genes obtained from each source, and the overlap between them. After removal of duplicates, 1,132 orthologous mouse cilia genes and predicted cilia genes remained.

Supplementary Figure S6.

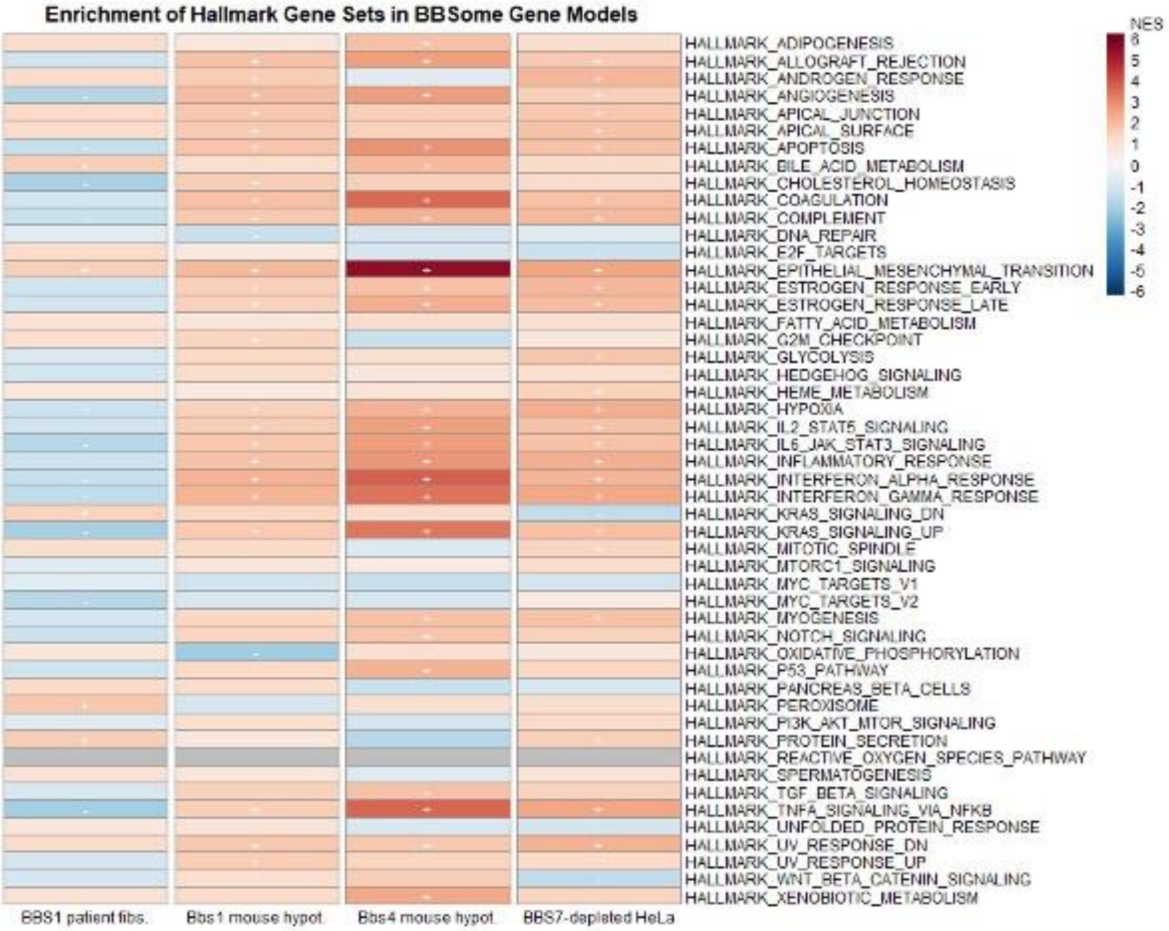

Supplement: Supplementary file 1 [file cells-12-02662-s001.zip › cells-2663995-supplementary.pdf]
