# Supplementary material for: De-Suppression of Mesenchymal Cell Identities and Variable Phenotypic Outcomes Associated with Knockout of Bbs1
Source: Cells. 2023 Nov 20;12(22):2662. doi: 10.3390/cells12222662 (PMC10670506; doi:10.3390/cells12222662)
Supplement: Supplementary file 1 [file cells-12-02662-s001.zip › Figure S7.pdf]

Supplementary Figure S7.

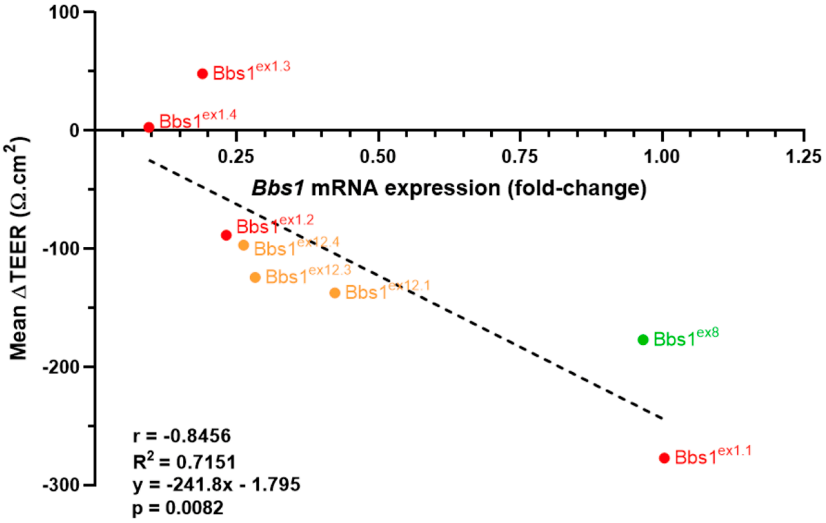

Correlation between *Bbs1* mRNA level and ΔTEER in eight *Bbs1*$^{-/-}$ clones including Bbs1ex12.1 and Bbs1ex12.4. There was a strong, negative correlation (r = -0.8456) that was statistically significant (p=0.0082). Points fit the model of linear regression well (R2= 0.7151). Each coloured point represents a single *Bbs1-/-* clone, which are labelled and coloured based on the sgRNA with which they were generated (red= sgRNA-1; green= sgRNA-8; orange= sgRNA-12).
